# A Gigantic Mistake

**Published:** 1899-08

**Authors:** 


### "A GIGANTIC MISTAKE."

If the steady elevation of the art of medicine to a science were due to a single factor—that factor is the germ theory of disease. The discovery of the cause of disease at once throws out conjecture and hypothesis and introduces exactness and precision.

It is not only the keystone of progress, but the hope of the future. It is the mainspring of preventive medicine; it has rendered possible the great achievements of surgery. It has strengthened and fortified and civilized by prolonging the life of man, and adding immeasurably to his comfort and well-being.

Even in this day of scornful infidelity there are few who doubted the truth of the doctrine of the bacteriological genesis of communicable disease. But now from rancorous unfaith or aimless iconoclasm or objectless pessimism George G. Bantock, in the *British Medical Journal*, declares that the modern doctrine of bacteriology is a gigantic mistake. The following is an abstract of his paper taken from *The Post-Graduate* for July:

"The author claims to have shown that the poisons of variola, vaccinia, and syphilis are not and cannot be the product of a bacillus; that Lœffler's bacillus is not a constant, and therefore cannot be the essential, element for the production of an attack of diphtheria; that the essential element in the case of gonorrhea is not the gonococcus; that the essential element in the case of typhoid fever is not the bacillus typhosus; that this bacillus cannot live but a few hours in ordinary sewage; that not a single specimen of this bacillus has ever been discovered in sewer air, and hence that typhoid fever cannot be attributed to it, because of its contained germs, that, in the cases of the epidemics at Maidstone and King's Lynn, there exists no proof of the contamination of the water by typhoidal matter, as indicated by the presence of the bacillus typhosus; that there is no evidence worthy of the name that tuberculosis is due to the ravages of the tubercle bacillus; that the comma bacillus cannot be regarded as the essential element in the production of an attack of cholera, and that the same can be said of the plague and its special bacillus; that the so-called pathogenic microorganisms are constantly found under conditions consistent with perfect health, and that in more than one notable instance they not only appear to, but actually do, exert a beneficent influence.

"All these things—which are facts, not opinions, capable of demonstration and proof—go to show, says the author, that the modern doctrine of bacteriology is a gigantic mistake; that we are already at the parting of the ways, and that it is safe to predict that, ere long, it will come to be recognized that these various bacilli play a beneficent *rôle* in the economy of Nature."

Such a man as Bantock—more's the pity—is entitled to a hearing, and his opinion bears weight and influence. It is hard to surmise how much harm belief in his opinions or facts, as he calls them, is calculated to do, but it is sufficient to say that the patient toil of decades, and the only real advance that medicine has made since Jenner's discovery, would be swept away.

It is comforting to know the modern doctrine of bacteriology can withstand any number of such assaults, and regard with absolute serenity such puny and pitifully impotent efforts to overturn it. It is well to remember also that nowhere has scientific progress met with greater obstruction than in England, as witness the constant fight againt vaccination that is being waged there; the attitude of the late Lawson Tait toward antiseptics; the strength of the anti-vivisection movement; and the shameful treatment of Havelock Ellis.

---

THOMAS OSMOND SUMMERS, of St. Louis, Mo., formerly editor of the *St. Louis Clinique*, and well known in connection with his work in yellow fever epidemics, committed suicide by shooting himself June 13. His death was somewhat theatrical. Before committing the deed he composed a sonnet bidding farewell to life, and then in the anatomical lecture-room of the college with which he was connected he "blew out the light within his brain."

---

LAWSON TAIT, F.R.C.S., died at Llandudno, Wales, June 17, at the age of 54. He was said to have been born out of wedlock, and to that fact was attributed much of the cynicism that characterized his life. He is principally known for his noteworthy additions to gynecologic surgery and for his polemics on the subject of asepsis as against the antiseptic doctrine of Lister.

---

DR. BARTON W. STONE, who formerly conducted Morningside Retreat at Nashville, has closed that institution and opened Beechhurst, a similar institution, at Louisville.

